# Cancer patterns and association with mortality and renal outcomes in non-dialysis dependent chronic kidney disease: a matched cohort study

**DOI:** 10.1186/s12882-019-1578-5

**Published:** 2019-10-22

**Authors:** Rajkumar Chinnadurai, Emma Flanagan, Gordon C. Jayson, Philip A. Kalra

**Affiliations:** 1 Department of Renal Medicine, Salford Royal NHS Foundation Trust, M6 8HD, Salford, UK; 2 Faculty of Biology, Medicine and Health, University of Manchester, Manchester, UK; 3 Information Management and Technology, Salford Royal NHS Foundation Trust, Salford, UK; 4 Manchester Cancer Research Centre, University of Manchester, Manchester, UK; 5 The Christie NHS Foundation Trust, Manchester, UK

**Keywords:** Cancer, Chronic kidney disease (CKD), All-cause mortality, End-stage renal disease, CKD progression, propensity score matching

## Abstract

**Background:**

Cancer in patients with chronic kidney disease (CKD) is an added burden to their overall morbidity and mortality. Cancer can be a cause or an effect of CKD. In CKD patients, a better understanding of cancer distribution and associations can aid in the proper planning of renal replacement therapy (RRT) and in the choice of chemotherapeutic agents, many of which are precluded in more advanced CKD. This study aims to investigate the distribution and the association of cancer with mortality, renal progression and RRT assignment in a non-dialysis dependent CKD cohort, few studies have investigated this in the past.

**Methods:**

The study was carried out on 2952 patients registered in the Salford Kidney Study (SKS) between October 2002 and December 2016. A comparative analysis was performed between 339 patients with a history of cancer (previous and current) and 2613 patients without cancer at recruitment. A propensity score matched cohort of 337 patients was derived from each group and used for analysis. Cox-regression models and Kaplan-Meier estimates were used to compare the association of cancer with mortality and end-stage renal disease (ESRD) outcomes. Linear regression analysis was applied to generate the annual rate of decline in estimated glomerular filtration rate (delta eGFR).

**Results:**

Of our cohort, 13.3% had a history of cancer at recruitment and the annual rate of de novo cancers in the non-cancer patients was 1.6%. Urogenital cancers including kidney and bladder, and prostate and testicle in males, ovary and uterus in females, were the most prevalent cancers (46%), as expected from the anatomical or physiological roles of these organs and relationship to nephrology. Over a median follow-up of 48 months, 1084 (36.7%) of patients died. All-cause mortality was higher in the previous and current cancer group (49.6% vs 35%, *p* < 0.001), primarily because of cancer-specific mortality. Multivariate Cox regression analysis showed a strong association of cancer with all-cause mortality (HR:1.41; 95%CI: 1.12–1.78; *p* = 0.004). There was no difference between the groups regarding reaching end-stage renal disease (26% in both groups) or the rate of decline in eGFR (− 0.97 for cancer vs − 0.93 mL/min/year for non-cancer, *p* = 0.93). RRT uptake was similar between the groups (17.2% vs 19.3%, *p* = 0.49).

**Conclusions:**

Cancer status proved to be an added burden and an independent risk factor for all-cause mortality but not for renal progression. CKD patients with a previous or current history of cancer should be assessed on a case by case basis in planning for renal replacement therapy options, and the presence of cancer should not be a limitation for RRT provision including transplantation.

## Introduction

Cancer is one of the leading causes of mortality and morbidity worldwide [1, 2]. In Europe, it is estimated that there will be 3.91 million new cases and 1.93 million deaths from cancer in 2018 [3]. Cancer prevalence is increasing in chronic kidney disease (CKD) patients due to improvements in life expectancy and better quality of care [4, 5]. Cancer and CKD are interrelated in many ways. Cancer patients develop CKD on account of the site of cancer, metastases, necessary chemotherapeutic treatments and management of related complications. Despite advances, chemotherapy-induced nephrotoxicity can be a significant barrier in the optimum management of cancer patients, more so in patients with CKD [6].

On the other hand, CKD is a risk factor for developing certain types of cancers such as liver and urogenital tract cancers [7–10]. Also, the presence of CKD in cancer patients is associated with a worse prognosis [11–13]. However, cardiovascular disease is still the leading cause of mortality in CKD patients [14].

In recent years, researchers have shown an increased interest in exploring the associations of cancer in CKD patients, leading to the emergence of the field of onco-nephrology [15–17]. Several inflammatory and oxidative stress mechanisms have been implicated in linking cancer with CKD [18, 19]. End-stage renal disease patients on dialysis or after transplantation are high-risk groups identified for cancer development due to uremia and immunodeficiency [20–23]. But, research on cancer and its impact on mortality and renal outcome is scarce in a Caucasian population with advanced CKD (CKD-3-5, not on dialysis) and the question of whether the presence of cancer accelerates the rate of progression of CKD is unexplored, which this study aims to address.

## Materials and methods

### Sampling

We conducted this study in the Salford Kidney Study (SKS) patients enrolled between October 2002 until December 2016. SKS, previously known as Chronic Renal Insufficiency Standard Implementation Study (CRISIS), is a large prospective cohort study recruiting non-dialysis CKD patients since 2002. Patient recruitment in the SKS has been described in previous published studies [24, 25]. In brief, any non-dialysis CKD patient above the age of 18 years and an eGFR less than 60 mL/min/1.73m^2^ referred to our tertiary renal service (1.55 million catchment population) is eligible to participate in this study. Upon recruitment, a questionnaire which includes patient demographic details, comorbidities including the history of malignancy and concurrent medications is completed. The patients are then followed up annually and comorbidities, cardiovascular events and hospital admissions are recorded. All patients provide informed consent, have blood results recorded upon registration and at annual follow up visits. The study has ethical approval for all observational data including mortality outcomes.

**Fig. 1:**
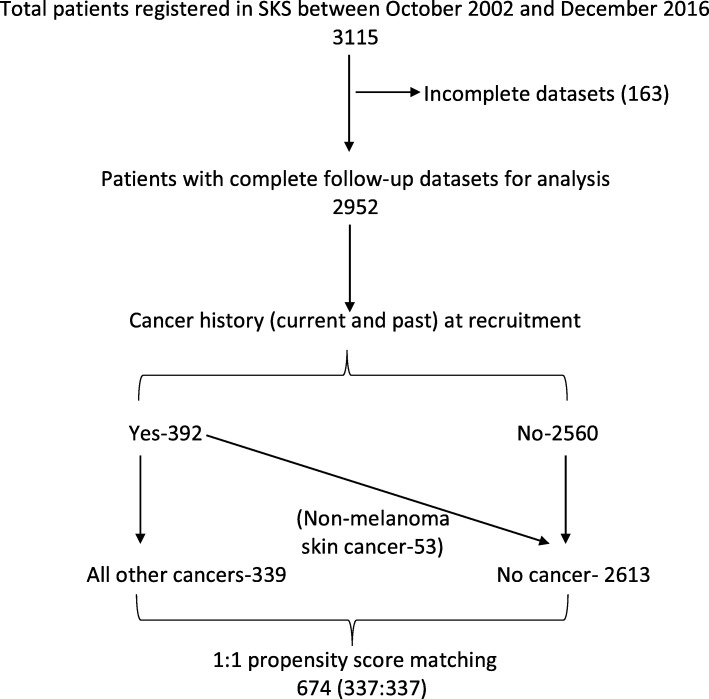
Flowchart of patient recruitment to the study

From a list of 3115 patients registered in the SKS over this 14-year period, 2952 patients with complete follow-up datasets were eligible. Of these, 392 reported having a history of cancer at study entry. All patients with a history of cancer (current and past) were included irrespective of the cancer site, stage and treatment status to allow calculation of the prevalence and incidence. In patients with multiple cancers, the first developed cancer was taken as the index cancer. Patients with non-melanoma skin (NMS) cancer were included in the no cancer group for further analysis. A matched cohort was generated using propensity score matching and was used for analysis. A flowchart of patient recruitment to the study is shown in Fig. 1.

### Data gathering

The date of SKS entry was used as the study baseline, and all patients were followed until they reached a study endpoint which was one of the following 1) commencement of renal replacement therapy (RRT) 2) death 3) end of analysis period - 31st December 2017 4) lost to follow up.

Data on patient baseline characteristics, baseline blood results, and date of death were gathered from the SKS database and electronic patient records (EPR). Initial blood results were those obtained at study entry or within 3 months. Smoking history was defined as a history of current or previous smoking, irrespective of the number of cigarettes smoked. Similarly, an alcohol history was defined as a history of current or past alcohol intake irrespective of the number of units. Non-fatal cardiovascular events (NFCVE) included a composite of an acute coronary syndrome, myocardial infarctions, non-fatal cardiac arrest, new diagnosis or hospital admission with congestive cardiac failure, cerebrovascular event and peripheral vascular disease. We defined end-stage renal disease (ESRD) as patients reaching RRT or eGFR ≤10 mL/min/1.73m^2^ in patients who opted for supportive care [26]. The cause of death was obtained from death certificates provided by the Office of National Statistics until the end of 2013 and thereafter from the Salford Royal electronic patient record only for those patients who died at our centre before December 2017. The eGFR values were calculated by the CKD epidemiology collaboration (CKD-EPI) formula [27].

### Statistical analysis

Propensity score matching was used to match patients with and without cancer to overcome the effects of selection bias and confounding factors [28]. The matching was undertaken by including all 20 baseline variables: age, gender, ethnicity, smoking status, alcohol consumption, systolic blood pressure (BP), diastolic BP, history of hypertension, diabetes mellitus, ischemic heart disease, myocardial infarction, congestive cardiac failure, cerebrovascular accident, peripheral vascular disease, chronic obstructive pulmonary disease, liver disease, use of statins, erythropoietin, renin-angiotensin blocking agents (ACE inhibitor or angiotensin receptor antagonist) and estimated glomerular filtration rate (eGFR). Matching was undertaken in a 1:1 ratio using the nearest neighbour method with the same propensity score. Comparative analyses of baseline characteristics, baseline blood results, mortality and renal replacement therapy were undertaken on both the total and propensity-matched samples.

Continuous non-parametric variables are presented as median (interquartile range), and the Mann–Whitney U test was used to compare statistical significance. Categorical data are expressed in percentages, and the Chi-square test was used for comparison.

The association of cancer with mortality and renal outcome (RRT) was calculated using univariate and multivariate Cox proportional hazards models to determine hazard ratios, 95% confidence intervals and statistical significance. To overcome the influence of competing risks, hazard ratios were derived by censoring at the competing event [29]. The multivariate models were developed by including the covariates based on the clinical plausibility of the causal association with outcome. A Kaplan-Meier (KM) curve was used to demonstrate cumulative survival. KM charts were also generated for all-cause mortality and RRT free survival by splitting the cancer group into three subgroups based on their cancer status at baseline; group A: concurrent cancer (diagnosed or treated in the previous 1 year), group B: history of cancer < 5 years (diagnosed or treated between > 1 year and < 5 years) and group C: cancer > 5 years (diagnosed or treated > 5 years ago). The association of cancer with CKD progression was computed using the rate of change of eGFR (eGFR slope) from study entry to study endpoint as assessed by the linear regression slope generated using serial serum creatinine measurements from outpatient clinic visits. Only patients with a minimum of three eGFR measurements were included in this model. The Mann–Whitney U test was used to compare the statistical significance between the groups. A *p*-value < 0.05 was considered statistically significant throughout the analysis. All statistical analysis was performed using IBM SPSS (Version 22), licenced to the University of Manchester. A competing risk analysis (CRA) for RRT, death and incident cancer between the groups was also performed using ‘cmprsk’ and ‘survival’ packages in R software, version 3.5.1 [30, 31]. A *p*-value for the CRA was calculated by the modified X^2^ statistic outlined in Gray, 1988 [32].

## Results

**Fig. 2:**
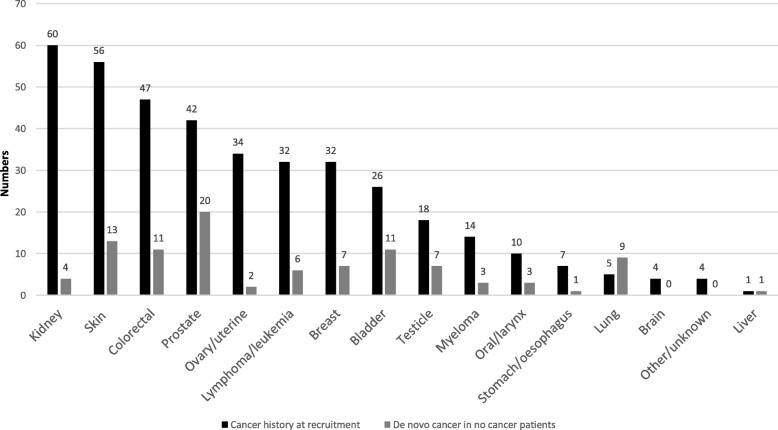
Cancer site distribution

A history of previous or current cancer was evident in 13.3% (392/2952) of our cohort, and the annual rate of de novo cancers in the non-cancer patients was 1.6%. The frequency distribution of various types of cancer is illustrated in Fig. 2. Urological cancers including prostate, kidney, bladder and ureter were the predominant sites of cancer in our cohort, contributing a third of the total number of cancers (32.6%). Of the 56 patients with skin cancers, 53 had non-melanomatous skin (NMS) cancer and three patients had low risk melanoma. All patients with skin cancer were treated curatively by local excision and had no recurrence during follow up. As NMS cancer patients were included in the no cancer group, the further analysis included 339 patients in the cancer group and 2613 patients in the no cancer group. The propensity score matched sample had 337 patients in each group.

The median age of our total cohort was 67 years with a predominance of males (62%) and Caucasians 96.1% (Table 1). Patients with a cancer history were older than those without cancer (71 versus 67 years, *p* < 0.001) and had a higher systolic blood pressure (140 versus 138 mmHg, *p* = 0.019). The groups were similar in distribution in terms of all cardiovascular risk factors at registration apart from the history of hypertension, which was more prevalent in patients without cancer. Other factors that were significantly different between the groups included the history of chronic obstructive pulmonary disease (COPD) and use of renin-angiotensin system (RAS) blockers. On review of initial blood results, the patients in the cancer group had a lower median haemoglobin (121 versus 123 g/l, *p* = 0.02), and eGFR (28.3 versus 30 mL/min/1.73m^2^, *p* = 0.018; Table 1). Although these differences noted are statistically different, they are probably not clinically significant. After propensity score matching the groups were very closely matched with no difference noted between the groups in any of the registration or biochemical characteristics.

**Table 1:** Comparison of baseline characteristics between cancer and no cancer patients in both total and matched samples

		Total sample			Propensity matched sample			
Variable	Total (2952)	Cancer (339)	No cancer (2613)	*p*-Value	Total (674)	Cancer (337)	No cancer (337)	*p*-Value
Age, years	67 (56–76)	71 (63–77)	67 (55–75)	**< 0.001**	71 (63–77)	71 (63–77)	71 (61–78)	0.79
Male	1834 (62.1%)	221 (65.2%)	1613 (61.7%)	0.22	434 (64.4%)	220 (65.3%)	214 (63.5%)	0.63
Caucasian	2837 (96.1%)	337 (99%)	2500 (95.67%)	**0.001**	670 (99.4%)	335 (99.4%)	335 (99.4%)	1.00
Smoking	1941 (65.8%)	228 (67.3%)	1713 (65.5%)	0.54	450 (66.8%)	227 (67.3%)	223 (66.2%)	0.74
Alcohol	1375 (46.5%)	144 (43%)	1231 (47%)	0.11	289 (42.9%)	144 (42.7%)	145 (43%)	0.94
BMI^a^, kg/m^2^	28 (24–32.6)	28.3 (24.7–32.7)	28 (24.6–32.6)	0.60	28 (24.8–31.6)	28.3 (24.7–32.8)	27.8 (24.8–31)	0.22
Systolic BP, mmHg	138 (124–153)	140 (126–156)	138 (124–152)	**0.019**	139 (126–155)	140 (126–156)	138 (125–152)	0.22
Diastolic BP, mmHg	75 (66–80)	74 (66–80)	75 (66–80)	0.58	74 (66–80)	74 (66–80)	75 (68–80)	0.41
Hypertension	2684 (90.9%)	295 (87%)	2389 (91.4%)	**0.008**	593 (87.9%)	295 (87.5%)	298 (88.4%)	0.72
DM	961 (37.6%)	108 (31.9%)	853 (32.6%)	0.77	215 (31.9%)	108 (32%)	107 (31.7%)	0.93
CVD	1269 (43%)	151 (44.5%)	1118 (42.8%)	0.54	298 (44.2%)	151 (44.8%)	147 (43.6%)	0.76
COPD	545 (18.5%)	44 (12.9%)	501 (23.7%)	**0.006**	87 (12.9%)	44 (13.1%)	43 (12.7%)	0.91
CLD	88 (2.9%)	7 (2.1%)	81 (3.1%)	0.29	13 (1.9%)	7 (2.1%)	6 (1.8%)	0.78
RAS blocker	1799 (61%)	162 (47.7%)	1637 (62.6%)	**< 0.001**	312 (46.3%)	162 (48.1%)	150 (44.5%)	0.35
Statin	1732 (58.7%)	194 (57.2%)	1538 (59%)	0.57	391 (58%)	194 (57.6%)	197 (58.5%)	0.82
ESA	379 (12.8%)	40 (11.8%)	339 (13%)	0.54	73 (10.8%)	40 (11.8%)	33 (9.8%)	0.39
HB, g/L	123 (112–135)	121 (109–131)	123 (112–135)	**0.02**	122 (112–133)	121 (110–131)	123 (113–134)	0.05
Albumin, g/L	43 (40–45)	43 (40–45)	43 (40–45)	0.30	43 (40–45)	43 (40–45)	43 (40–45)	0.50
ALP, Units/L	82 (66–104)	85.5 (68–107)	82 (65–103)	**0.04**	84 (67–107)	85.5 (68–107)	85.5 (69–108.5)	0.99
Calcium, mmol/L	2.3 (2.2–2.4)	2.32 (2.2–2.4)	2.30 (2.2–2.4)	0.17	2.31 (2.2–2.4)	2.32 (2.2–2.4)	2.30 (2.2–2.4)	0.55
Phosphate, mmol/L	1.12 (0.97–1.3)	1.10 (0.95–1.3)	1.12 (0.98–1.3)	0.35	1.11 (0.96–1.3)	1.10 (0.95–1.3)	1.12 (0.97–1.3)	0.49
uPCR^a^, g/mol	31.5 (13–104)	34.23 (13.2–87)	31.2 (13–107)	0.49	33.3 (13.1–90.7)	33.7 (13.1–85)	32.1 (13.5–94.7)	0.64
Creatinine, micromol/L	182 (135–256)	188 (141–262)	179 (135–254)	0.16	185 (138–262)	187 (141–261)	183 (135–263)	0.53
eGFR, mL/min/1.73m^2^	30 (19.7–42.5)	28.3 (18.3–39.3)	30 (19.8–43)	**0.02**	29.3 (18.3–40.5)	28.7 (18.5–39.4)	29.6 (18–40.9)	0.60

a-BMI missing in 530/2952 of total sample and in 124/674 of matched sample. b-missing uPCR values in 292 patients of total sample and 75 patients of matched sample

BMI-body mass index, BP-blood pressure, DM-diabetes mellitus, CVD- cardio vascular disease (includes at least one of the following- ischemic heart disease, myocardial infarction, congestive cardiac failure, cerebrovascular accident, and peripheral vascular disease), COPD-chronic obstructive pulmonary disease, CLD-chronic liver disease, RAS-renin-angiotensin system blocker, ESA-erythropoietin stimulating agents, HB-haemoglobin, ALP-alkaline phosphatase, uPCR-urine protein creatinine ratio, eGFR - estimated glomerular filtration rate calculated by CKD-EPI equation

Continuous variables are expressed as median (interquartile range) and *p*-Value by Man-Whitney U test

Categorical variables are expressed as number (%) and *p*-Value by Chi-Square test. Statistically significant p-values are displayed in bold (i.e. *p* < 0.05)

**Fig. 3:**
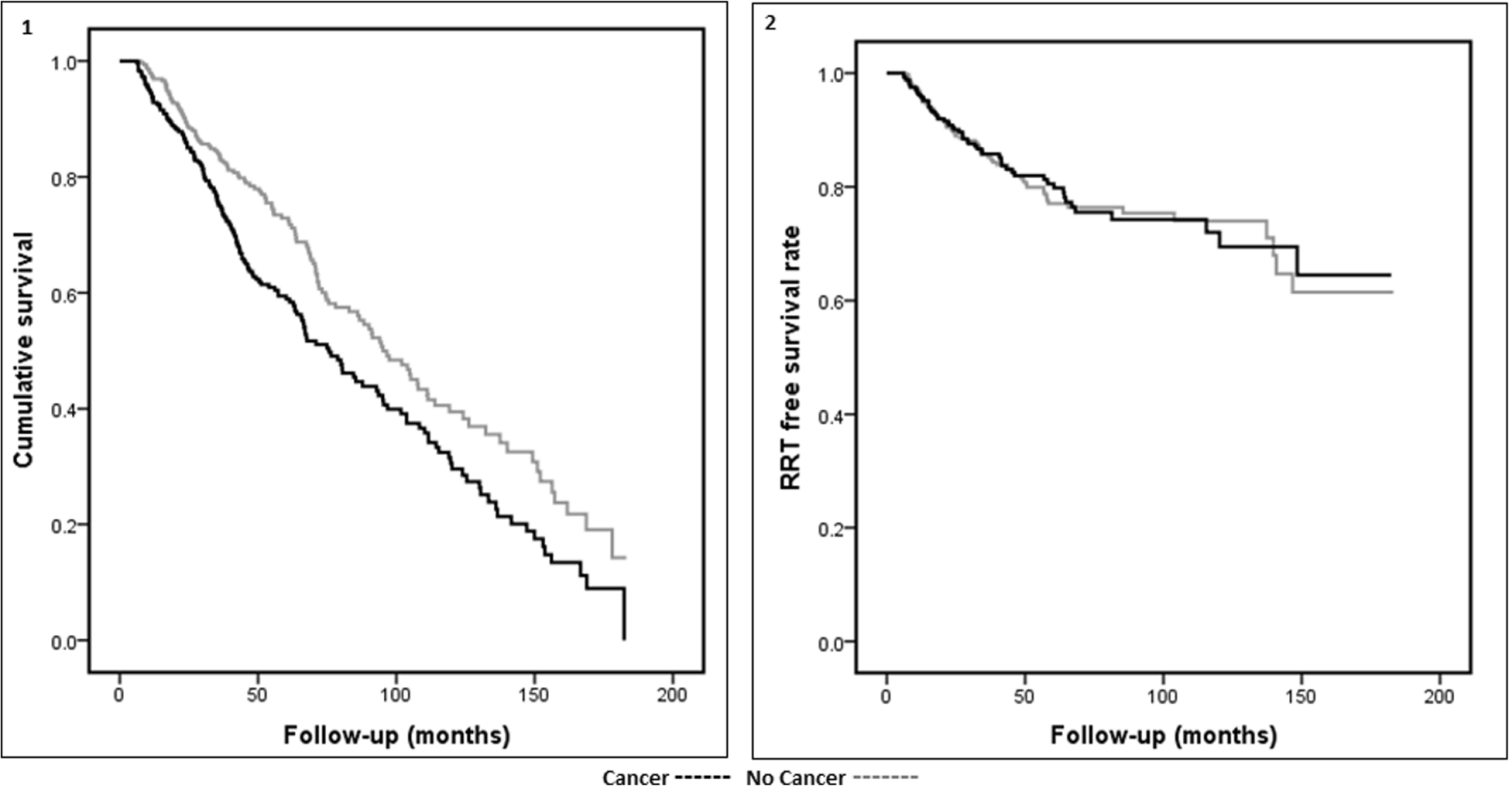
Kaplan-Meier curve in the matched sample (panel-1: all-cause mortality, panel-2: renal replacement therapy (RRT) free survival)

During a median follow up of 48 months, 1084 (36.7%) patients died. The all-cause mortality rate was significantly higher in the cancer group even in the matched groups (49.3% versus 38.3%, *p* = 0.004). The cause of death data was available only in 474 of the 1084 patients who died (44%). Cancer-related death was significantly higher in the cancer group than the no cancer group (26.3% versus 13.5%, *p* = 0.005). Deaths due to cardiovascular disease and infections were similar in the groups. In the matched sample, age at death was significantly less in patients with cancer (79 versus 80.5 years, *p* = 0.03) (Table 2). This survival difference is also illustrated in the KM survival curve (log-rank; *p* = 0.002) (Fig. 3).

**Table 2:** Comparison of mortality and renal outcomes between cancer and no cancer patients in both total and matched sample

		Total sample				Propensity	matched sample	
Variable	Total (2952)	Cancer (339)	No cancer (2613)	*p*-Value	Total (674)	Cancer (337)	No cancer (337)	*p*-Value
Follow-up, months	48 (25–79)	40.3 (20.5–68)	48.2 (25–79)	**0.001**	43 (21–75)	40.6 (21–68)	47.1 (21.6–78.5)	0.09
Age at death, years	78.7 (72–84)	79 (72.4–84.7)	78.5 (72.1–84.4)	0.88	79 (72–84)	79 (72–84)	80.5 (75–86)	**0.03**
All-cause mortality	1084 (36.7%)	168 (49.6%)	916 (35%)	**< 0.001**	295 (43.8%)	166 (49.3%)	129 (38.3%)	**0.004**
Cause of death (cancer)	74/474 (15.6%)	20/76 (26.3%)	54/398 (13.5%)	**0.005**	26/128 (20.3%)	20/76 (26.3%)	6/52 (11.5%)	**0.04**
Cause of death (CVD)	154/474 (32.5%)	20/76 (26.3%)	134/398 (33.7%)	0.21	33/128 (25.7%)	20/76 (26.3%)	13/52 (25%)	0.87
Cause of death (infection)	124/474 (26.1%)	16/76 (21%)	108/398 (27.1%)	0.27	30/128 (23.4%)	16/76 (21%)	14/52 (27%)	0.44
NFCVE	282 (9.55%)	30 (8.9%)	252 (9.6%)	0.64	52 (7.71%)	30 (8.9%)	22 (6.5%)	0.25
ESRD (RRT+ eGFR <or = 10)	855 (29.9%)	87 (25.7%)	768 (29.4%)	0.16	174 (25.8%)	87 (25.8%)	87 (25.8%)	1.00
CC	233 (7.9%)	29 (8.6%)	204 (7.8%)	0.18	51 (7.6%)	29 (8.6%)	22 (6.5%)	0.24
RRT	622 (21%)	58 (17.1%)	564 (21.6%)	0.06	123 (18.25%)	58 (17.2%)	65 (19.3%)	0.49
First start RRT modality (TX)	97/622 (15.5%)	3/58 (5.17%)	94/564 (16.6%)	**0.02**	10/123 (8.1%)	3/58 (5.2%)	7/65 (10.7%)	0.26
First start RRT modality (HD)	323/622 (51.9%)	40/58 (68.9%)	283/564 (50.2%)	**0.006**	78/123 (63.4%)	40/58 (68.9%)	38/65 (58.5%)	0.23
First start RRT modality (PD)	202/622 (32.5%)	15/58 (25.9%)	187/564 (33.2%)	0.26	35/123 (28.5%)	15/58 (25.8%)	20/65 (30.8%)	0.55

Cause of death represents 1a cause of death in death certificate. Cause of death available only in 474/1084 patients of the total sample (76/168 in cancer group and 398/916 in no cancer group) and 128/295 of matched sample (76/166 in cancer group and 52/129 in no cancer group). CVD-cardiovascular disease

*NFCVE* non-fatal cardiovascular events, *ESRD* end-stage renal disease, *eGFR* estimated glomerular filtration rate, *RRT* renal replacement therapy, *CC* conservative care, *TX* renal transplant, *PD* peritoneal dialysis, *HD* haemodialysis. Continuous variables are expressed as median (interquartile range) and *p*-Value by Man-Whitney U test

Categorical variables are expressed as number (%) and *p*-Value by Chi-square test. Statistically significant p-values are displayed in bold (i.e. *p* < 0.05)

There were a total of 282 NFCVE reported during the follow-up period and the events were similar between the groups in both the overall and propensity-matched analyses. On review of renal outcomes, 30% of patients reached ESRD. The median age of the patients at the time of commencement of haemodialysis was 63 years, peritoneal dialysis was 62 years, and a transplant was 52 years. The RRT uptake was similar in the two groups in the matched sample (17.2% versus 19.3%, *p* = 0.49), also shown in KM curve (log-rank; *p* = 0.93; Fig. 3). The first RRT modality was predominantly haemodialysis in the cancer group (68.9%). More patients in the no cancer group received a renal transplant compared to the cancer group (42.9% versus 15.5%, *p* < 0.001), but these differences were not observed once the groups were matched (Table 2).

In Cox-proportional hazard models, a cancer history at baseline showed strong association with all-cause mortality in the univariate model (HR: 1.64; 95%CI: 1.39–1.93; *p* < 0.001). The strength of association persisted in all four multivariate models developed by adding covariates in a stepwise manner. In a multivariate model (model 4) in the matched sample which included all initial clinical and demographic variables, cardiovascular risk factors and eGFR, the presence of cancer showed an independent association with all-cause mortality (HR: 1.41; 95%CI: 1.12–1.78; *p* = 0.004) (Table 3).

**Table 3:** Association of cancer with all-cause mortality and end-stage renal disease (Cox regression models)

	Total sample		Propensity matched sample	
	HR (95% CI)	*p*-Value	HR (95% CI)	*p*-Value
All-cause mortality				
Univariate model	1.64 (1.39–1.93)	**< 0.001**	1.43 (1.13–1.80)	**0.002**
Multivariate model 1	1.40 (1.19–1.66)	**< 0.001**	1.37 (1.09–1.73)	**0.006**
Multivariate model 2	1.43 (1.21–1.69)	**< 0.001**	1.40 (1.11–1.76)	**0.004**
Multivariate model 3	1.49 (1.26–1.76)	**< 0.001**	1.40 (1.12–1.77)	**0.004**
Multivariate model 4	1.45 (1.22–1.70)	**< 0.001**	1.41 (1.12–1.78)	**0.004**
End-stage renal disease				
Univariate model	1.01 (0.81–1.25)	0.97	1.10 (0.82–1.48)	0.52

Multivariate Model 1: Adjusted for age, gender, ethnicity

Multivariate Model 2: Adjusted for all covariates of model 1 plus smoking, alcohol, hypertension, diabetes mellitus

Multivariate Model 3: Adjusted for all covariates of model 2 plus ischaemic heart disease, myocardial infarction, congestive cardiac failure, cerebro vascular accident, peripheral vascular disease

Multivariate Model 4: Adjusted for all covariates of model 3 plus estimated glomerular filtration rate (CKD-EPI)

HR-Hazard ratio, CI-Confidence interval. Statistically significant p-values are displayed in bold (i.e. *p* < 0.05)

There was no clear correlation observed between cancer status and reaching end-stage renal disease (HR: 1.01; 95%CI: 0.81–1.25; *p* = 0.97) (Table 3). Also, CKD progression as determined by the rate of decline in eGFR was not different between the groups (cancer patients − 0.96 versus − 1.24 mL/min/1.73m^2^/year) (Table 4). Similar observations were noted in the propensity matched sample with regards to the renal outcomes. In a sub-analysis, urological cancers (kidney, bladder and prostate) were not associated with an increased risk of RRT either compared to non-cancer patients or those with cancer from other causes.

**Table 4:** Rate of decline of eGFR (CKD progression)

	Total sample			Propensity matched	sample	
Variable	Cancer (332)	No cancer (2532)	*p*-Value	Cancer (328)	No cancer (328)	*p*-Value
eGFR slope mL/min/1.73m^2^/year	−0.96 (−2.8 to 0.91)	−1.24 (−3.2 to 0.58)	0.07	− 0.97 (− 2.9 to 0.91)	−0.93 (− 2.71 to 0.84)	0.93

eGFR (CKD EPI)- estimated glomerular filtration rate calculated by CKD-EPI equation

Values expressed as median (interquartile range). *p*-Value by Mann-Whitney U test

Rate calculated only from patients with minimum three eGFR results

Competing risk models developed on the matched sample showed the probability of death was higher in the cancer group at 5, 10 and 15 years. At five years the probability of death was 36% in the cancer group compared to 23% in the no-cancer group, the difference being statistically significant (*p* = 0.001). There was no difference observed in the cumulative probability for renal replacement therapy or incident cancer between the groups (cancer vs no-cancer) during follow-up (Table 5) (Additional file 1: Figure S1).

**Table 5:** Cumulative incidence probability for death and renal replacement therapy between the groups (cancer vs no cancer) in the matched sample in a competing risk analysis

Years from study entry	Groups	Death	Renal replacement therapy
5	Cancer	0.36	0.16
	No cancer	0.23	0.20
10	Cancer	0.57	0.19
	No cancer	0.47	0.21
15	Cancer	0.70	0.20
	No cancer	0.63	0.25
	***p*** **-Value**	**0.001**	0.39

*p*-Value by modified X^2^ test by Gray. Statistically significant p-values are displayed in bold (i.e. *p* < 0.05)

The sub-analysis of 337 patients (matched sample) who had a previous or current cancer history at registration showed that 58 patients were in group A, 119 in group B and 160 in group C. A higher proportion of patients in group A (56.9%) and B (56.3%) died compared to that of group C (41%) (A vs C, *p* = 0.04). Death from cancer was more likely in group A (35.3% vs 26.7 and 17.2% in groups B and C, respectively) while death from cardiovascular disease was more prevalent in group C (34.5%). RRT uptake was higher in group C (23% vs 12.1% in group A) with a greater proportion of ESRD patients receiving transplants in groups B and C (11.4 and 12% vs 5.3% in group A) (Table 6).

**Table 6:** Comparison of outcomes between the groups split based on date of cancer occurrence prior to recruitment in matched sample

Outcome	No cancer (337)	Concurrent history of cancer < 1 year Group A (58)	Cancer history > 1 year and < 5 years Group B (119)	Cancer history > 5 years Group C (160)	*p*-Value Group A vs Group C
Death	129 (38.3%)	33 (56.9%)	67 (56.3%)	66 (41.2%)	**0.04**
Cancer death	6/52 (11.5%)	6/17 (35.3%)	8/30 (26.7%)	5/29 (17.2%)	0.16
CVD death	13/52 (25%)	2/17 (11.8%)	9/30 (30%)	10/29 (34.5%)	0.09
ESRD	87 (25.8%)	19 (32.8%)	24 (20.1%)	44 (27.5%)	0.45
RRT	65 (19.3%)	7 (12.1%)	14 (11.8%)	37 (23.1%)	0.07
Transplants	7/65 (10.8%)	0/7	1/14 (7.1%)	2/37 (7.4%)	> 0.05^a^

Cause of death data was available only in 52/129 of the no cancer group, 17/33 of group A, 30/67 of group B and 29/66 of group C. *CVD* cardiovascular disease, *ESRD* end-stage renal disease, *RRT* renal replacement therapy. *p*-Value by Chi-square test, a-*p*-Value by Fisher Exact test. Statistically significant p-values are displayed in bold (i.e. *p* < 0.05)

The KM analysis of these subgroups (A, B & C) showed a distinction between the different cancer history and survival outcomes. As expected, patients with concurrent cancer at registration had the worst outcome (log-rank, *p*-Value = 0.001) (Additional file 1: Figure S2). Although the KM graphs appear to be different between the groups for RRT free survival, this did not reach statistical significance (log-rank, *p*-Value = 0.079; (Additional file 1: Figure S3). In a KM chart of outcomes of patients with different cancer sites in the matched sample there was an overlap of the survival pattern of certain cancer sites (breast/haematological) with the non-cancer group, but there was a clear distinction and poor survival noted for other cancer sites (lung, gastrointestinal tract, urogenital, other) (log-rank, *p* = 0.001) (Additional file 1: Figure S4).

## Discussion

This is one of the largest studies examining cancer and its associations in a Caucasian cohort with advanced CKD. The study describes the pattern of cancer distribution in a non-dialysis CKD population and its impact on mortality and renal outcomes. The concurrent or previous cancer history of 13.3% and annual incidence of 1.6% were similar to those observed in other CKD cohorts [33, 34]. Cancer site distribution was comparable to that seen in other CKD groups, with an understandably high prevalence of urological malignancy; the most prevalent and incident (35.7%) cancers [35]. Surgical treatment of kidney and urothelial cancers would have led to the referral to nephrology services in many cases. However, the prevalence of liver cancer was noted to be low in our cohort (0.26%), potentially reflecting a low prevalence of chronic liver disease (2.9%) in the population.

In our analysis, patients with non-melanoma skin (NMS) cancers were included in the no cancer group as the cancer was localised in all patients, and all had curative treatment with no recurrence. The 10-year survival rate for NMS cancer with treatment is similar to people without cancer in the general population [36]. Patients in the cancer group were significantly older, an age-related association with cancer also noted in the general population [37]. It is well known that CKD is a pro-inflammatory state and chronic inflammation with ageing has been linked with tumorigenesis [38]. The CKD-EPI equation was used in the calculation of eGFR in our analysis due to the increasing evidence to support the use of this formula in cancer patients [39]. In the cancer group, median haemoglobin was marginally low and alkaline phosphatase was high, both of these observations probably a reflection of a lower eGFR (28.3 vs 30 mL/min; *p* = 0.01). However, these differences in baseline and biochemical characteristics were not observed once the groups were propensity matched.

In the matched samples, age at death was significantly lower in patients with cancer (79 versus 80.5 years, *p* = 0.03). The KM estimate verified this difference in survival (log-rank; *p* = 0.001). All-cause mortality was high in cancer patients, predominantly influenced by more cancer-specific mortality (26.3% versus 13.5%, *p* = 0.005). The presence of CKD as comorbidity has been shown to be a risk factor for mortality in cancer patients [40]. Despite this, the cardiovascular disease (CVD) and infection-related mortality burden were similar in the two groups. It was also interesting to note that even in the cancer group the CVD related mortality was equivalent to the cancer-related mortality (26.3%) and there was no significant difference in the number of NFCVE reported between the groups.

Both univariate and multivariate Cox-regression models have consistently shown the presence of cancer as a strong independent risk factor for all-cause mortality. In the model-3 of the multivariate Cox-regression analysis which was adjusted for baseline variables including all cardiovascular risk factors, cancer showed a HR: 1.49 (CI: 1.26–1.76; *p* < 0.001) (Table 3). In the Japanese CKD cohort study of Tanaka et al. cancer was associated with cancer-associated but not all-cause mortality. However, our observations involved a considerably larger sample size than the Japanese study (2952 versus 515) [34].

The proportion of patients reaching ESRD were similar in the two groups. In the unmatched analysis, proportionately more cancer patients started haemodialysis with fewer transplants, but once propensity matching was undertaken these differences disappeared. There was no clear correlation between cancer status and reaching end-stage renal disease in the univariate model hence further multivariate models were not generated. Also, in linear regression analysis, the presence of cancer was not associated with accelerated rate of progression of CKD. The overall rate of decline in eGFR in our cohort was similar to that seen in other European cohorts [41]. The competing risk models inferred similar results; higher probability of death in the cancer group, with no difference in RRT uptake or incident cancer between the groups. To our knowledge this is the first study in the literature evaluating the association of baseline cancer status with renal outcome and progression in a non-dialysis dependent European CKD cohort. It was evident from the subgroup analysis that survival outcome in patients with previous cancer was improved with a greater period of cancer in remission, inferring that these patients can be carefully considered for all RRT options including transplantation.

Our study does have some limitations not least the observational nature of the methodology. The missing cause of death data restricted our strength of conclusions in the matched sample due to small numbers. We were not able to account for cancer treatment status or stage at the time of recruitment into the cohort, a deficit which could introduce bias into the interpretation of the findings. As our study only included patients who volunteered to participate in the cohort the generalisation of the age-adjusted cancer incident rates to the general CKD population is necessarily limited. Despite these, the study’s strengths included a robust database with a large sample size, propensity matching and accurate follow-up data.

## Conclusions

Our study has shown that cancer is a strong and independent risk factor for all-cause mortality in advanced CKD. Cardiovascular disease is still a leading cause of death in CKD patients, even in patients with cancer. Baseline cancer status did not accelerate the rate of progression of CKD. Our study findings imply that CKD patients with cancer have to be assessed on a case by case basis in planning for renal replacement therapy options. With advancing cancer management options, the presence of cancer should not be a limitation for RRT provision, including transplantation in selected cases.

## Supplementary information

**Additional file 1: Figure S1.** Cumulative incidence probability for death and renal replacement therapy (RRT) between the groups (cancer vs no cancer) in the matched sample. **Figure S2.** Kaplan-Meier curve for all-cause mortality in the matched sample (comparison between groups split based on date of cancer occurrence prior to recruitment). **Figure S3.** Kaplan-Meier curve for renal replacement therapy (RRT) free survival in the matched sample (comparison between groups split based on date of cancer occurrence prior to recruitment). **Figure S4.** Kaplan-Meier curve for all-cause mortality in the matched sample (comparison between groups split based on cancer sites with no cancer).

## Data Availability

This study derives data from a precious long-standing database in which data has been meticulously collected over 20 years. The authors are shortly planning to perform further analyses from the data, and these would be compromised if the database were made publically available but are available from the corresponding author on reasonable request.

## Abbreviations

BP: Blood pressure
CI: Confidence interval
CKD: Chronic kidney disease
CRA: Competing risk analysis
CVD: Cardiovascular disease
eGFR: estimated glomerular filtration rate
EPR: Electronic patient records
ESRD: End-stage renal disease
HR: Hazard ratio
KM: Kaplan Meier
NFCVE: Non-fatal cardiovascular events
NMS: Non-melanomatous skin
RAS: Renin-angiotensin system
RRT: Renal replacement therapy
SKS: Salford Kidney Study
