# Supplementary material for: Cancer patterns and association with mortality and renal outcomes in non-dialysis dependent chronic kidney disease: a matched cohort study
Source: BMC Nephrol. 2019 Oct 22;20:380. doi: 10.1186/s12882-019-1578-5 (PMC6805476; doi:10.1186/s12882-019-1578-5)

**Figure S1. Cumulative incidence probability for death and renal replacement therapy (RRT) between the groups (cancer vs no cancer) in the matched sample**

**
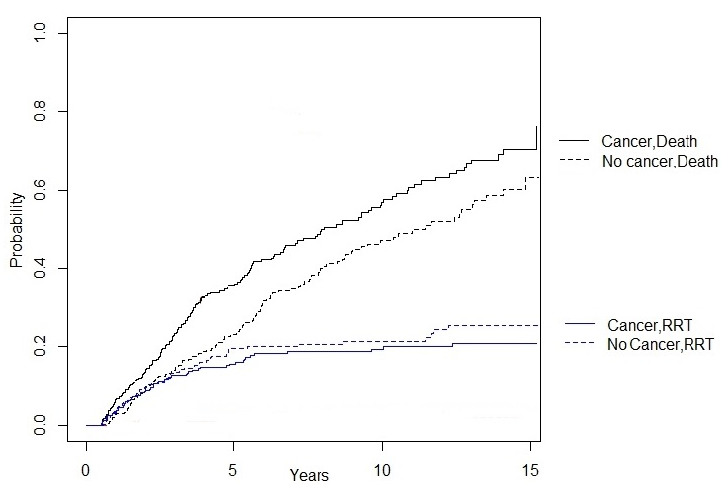
**

**Figure S2. Kaplan-Meier curve for all-cause mortality in the matched sample (comparison between groups split based on date of cancer occurrence prior to recruitment)**


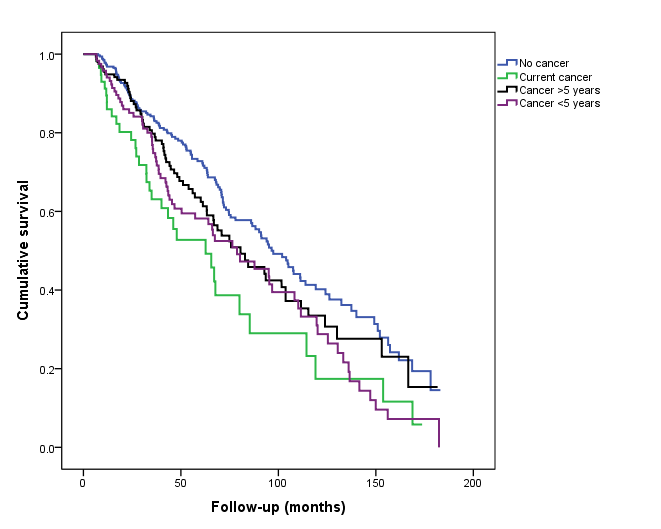


**Figure S3. Kaplan-Meier curve for renal replacement therapy (RRT) free survival in the matched sample (comparison between groups split based on date of cancer occurrence prior to recruitment)**


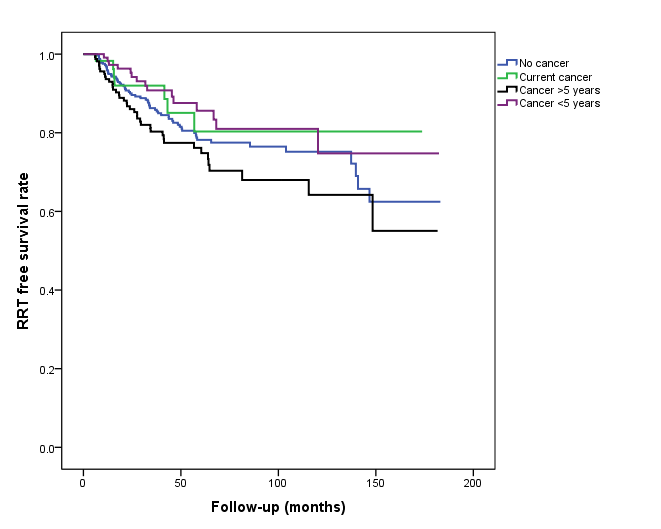


**Figure S4. Kaplan-Meier curve for all-cause mortality in the matched sample (comparison between groups split based on cancer sites with no cancer)**


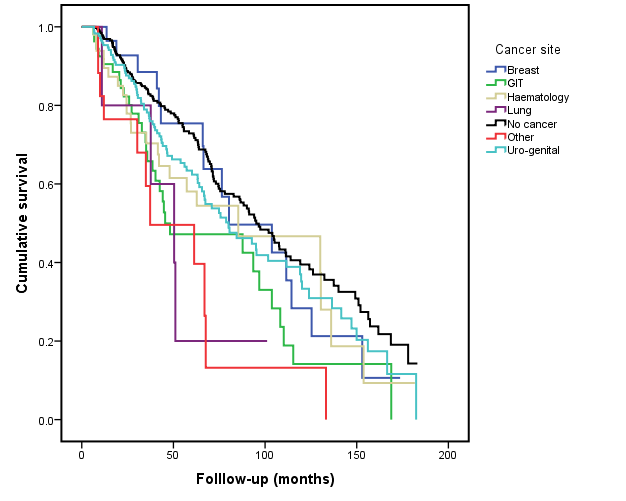

Supplement: Supplementary file 1 — Additional file 1: Figure S1. Cumulative incidence probability for death and renal replacement therapy (RRT) between the groups (cancer vs no cancer) in the matched sample. Figure S2. Kaplan-Meier curve for all-cause mortality in the matched sample (comparison between groups split based on date of cancer occurrence prior to recruitment). Figure S3. Kaplan-Meier curve for renal replacement therapy (RRT) free survival in the matched sample (comparison between groups split based on date of cancer occurrence prior to recruitment). Figure S4. Kaplan-Meier curve for all-cause mortality in the matched sample (comparison between groups split based on cancer sites with no cancer). [file 12882_2019_1578_MOESM1_ESM.docx]
